# Controlled Ice Nucleation With a Sand-PDMS Film Device Enhances Cryopreservation of Mouse Preantral Ovarian Follicles

**DOI:** 10.1115/1.4066445

**Published:** 2024-12-01

**Authors:** Samantha Stewart, Alisa White, Wenquan Ou, Wei Liu, Jennifer Nagashima, Nucharin Songsasen, Xiaoming He

**Affiliations:** Fischell Department of Bioengineering, University of Maryland, College Park, MD 20742; Center for Species Survival, Smithsonian National Zoo and Conservation Biology Institute, 1500 Remount Road, Front Royal, VA 22630; Fischell Department of Bioengineering, University of Maryland, College Park, MD 20742; Robert E. Fischell Institute for Biomedical Devices, University of Maryland, College Park, MD 20742; Marlene and Stewart Greenebaum Comprehensive Cancer Center, University of Maryland, Baltimore, MD 21201

**Keywords:** ice-seeding, slow freezing, sand, PDMS, tissue, fertility, preservation

## Abstract

Ovarian follicle cryopreservation is a promising strategy for fertility preservation; however, cryopreservation protocols have room for improvement to maximize post-thaw follicle viability and quality. Current slow-freezing protocols use either manual ice-seeding in combination with expensive programmable-rate freezers or other clinically incompatible ice initiators to control the ice-seeding temperature in the extracellular solution, a critical parameter that impacts post-cryopreservation cell/tissue quality. Previously, sand has been shown to be an excellent, biocompatible ice initiator, and its use in cryopreservation of human induced pluripotent stem cells enables high cell viability and quality after cryopreservation. This study applies sand as an ice initiator to cryopreserve multicellular microtissue, preantral ovarian follicles, using a simple slow-freezing protocol in the mouse model. Ovarian follicles cryopreserved using the sand partially embedded in polydimethylsiloxane (PDMS) film to seed ice in the extracellular solution exhibit healthy morphology, high viability, and the ability to grow similarly to fresh follicles in culture post-thaw. This sand-based cryopreservation strategy can facilitate convenient ovarian follicle cryopreservation using simple equipment, and this study further demonstrates the translatability of this strategy to not only single cells but also multicellular tissues.

## Graphical abstract

**Figure:**
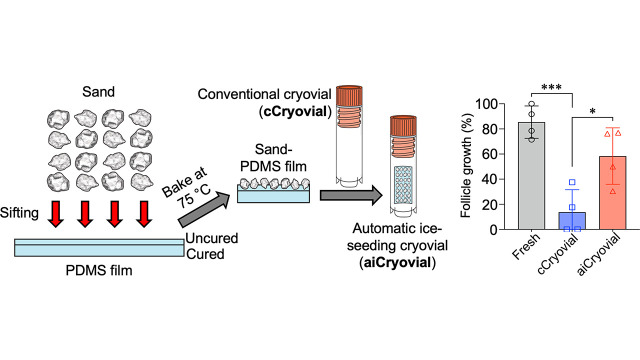

## Introduction

The demand of fertility preservation for medical and personal reasons has dramatically increased in recent years [1]. Mature oocyte or embryo cryopreservation is currently the most widely explored techniques for fertility preservation [2]. This method requires at least one cycle of controlled hormonal ovarian stimulation to collect the mature oocytes for either immediate banking or to produce embryos [3], which is not suitable for prepubertal patients, patients with hormone-responsive diseases, or patients who cannot delay disease treatment to undergo hormonal stimulation [4]. Embryo freezing not only faces potential legal, moral, ethical, and religious concerns [5], but does not allow for reproductive autonomy for the patient—they must rely on a sperm source, either from a partner or donor, to preserve their fertility [6]. Ovarian tissue cryopreservation is a promising option for prepubertal cancer patients and postpubertal cancer patients who cannot undergo controlled ovarian stimulation [7]. However, for some patients, this approach carries the risk of re-introducing malignant cells that could have infiltrated the ovarian tissue prior to cryopreservation back into patients and causing cancer recurrence [8–10]. To address these challenges, isolation and cryopreservation of ovarian follicles, which are not vascularized tissues that can contain malignant cells, has been explored.

Although results of cryopreserving isolated ovarian follicles using slow-freezing protocols have shown encouraging success [11,12], the method still faces technical challenges that need to be addressed. For example, many slow-freezing methods require the use of expensive programmable-rate freezers and manual ice-seeding in the sample at high subzero temperatures [13–15]. Uncontrolled, spontaneous ice formation is a stochastic event that can occur at temperatures below −15 °C, which may be detrimental and often lethal to cells [16–20]. During slow cooling of cells in aqueous samples, ice first nucleates and grows in the extracellular space [21–23]. The lower the temperature of extracellular ice formation, the more ice embryos (tiny aggregates of crystalline water molecules that spontaneously form) can initiate in the sample. This leads to fine ice crystals that can cause physical damage to the cell membrane and induce intracellular ice formation of deeply supercooled intracellular water, which is highly lethal to cells [17–20]. Extracellular ice formation at low subzero temperatures can also cause osmotic shock-associated damage to the cells, as the sudden ice formation induces a rapid increase in the local osmolality of the extracellular solution surrounding the growing ice crystals [20]. Conversely, when ice nucleates in a controlled fashion at high subzero temperatures, fewer ice embryos form in the given sample. These extracellular ice embryos gradually grow into large ice crystals during subsequent cooling, which may allow more time for intracellular water to diffuse out of cells, minimizing both intracellular ice formation and osmotic shock.

Several methods have been used to control the instance of ice-seeding in samples during cryopreservation to improve cryopreservation outcomes [24,25], like seeding ice manually with precooled metal tools [20,26] or adding ice nucleators like the bacteria *Pseudomonas syringae* into the sample [27–30]. These methods can be lengthy, difficult to standardize, and hard to bring into compliance with current good manufacturing practice (cGMP), making it difficult to apply these ice-seeding methods to the cryopreservation of clinical-grade cells [25]. Previously, we used a natural, biocompatible material—sand—consistently and reliably seed ice above −10 °C (measured in pure water) for slow-freezing cryopreservation [31]. This controlled seeding enabled serum-free cryopreservation of human induced pluripotent stem cells with high viability, pluripotency, using a low concentration of cryoprotective agents (CPAs). However, it has not been tested for enhancing the cryopreservation of multicellular tissues.

In this study, we used this sand-based efficient ice-seeding method to assist in cryopreservation of ovarian follicles, a similarly stress-sensitive tissue. Ovarian follicles cryopreserved using the sand particles partially embedded in a polydimethylsiloxane (PDMS) film that is immobilized on the wall of the cryovial to nucleate ice at high subzero temperatures, show higher immediate viability than that of ovarian follicles cryopreserved without ice-seeding. Furthermore, they grow similarly to freshly isolated follicles for 9 days of in vitro culture. Incorporating sand in the sample as an easy, biocompatible ice nucleator, can simplify the cryopreservation process of ovarian follicles while still maintaining follicle viability and capacity to grow in culture. This can eliminate the need for expensive programmable-rate freezers, lengthy and troublesome manual ice-seeding procedures, and concerns about non-cGMP.

## Materials and Methods

All cell culture materials (media, L-glutamine, and penicillin/streptomycin) were purchased from ThermoFisher (Waltham, MA) unless otherwise indicated. All chemicals were purchased from Sigma-Aldrich (St. Louis, MO) unless otherwise indicated.

### Fabrication of Sand-Polydimethylsiloxane Film Device.

The sand-PDMS film devices were fabricated according to a previously reported protocol [31]. Briefly, sand was purchased from Walmart (Landover Hills, MD) and rinsed under running tap water overnight in a 100 mL beaker after 10 min of agitation with a stir bar. The sand was then washed twice with 50 mL of de-ionized water, autoclaved at 121 °C for 30 min, and baked at 75 °C for 6 h to dry. To form the film, PDMS (Dow, Midland, MI) prepolymer was mixed with its curing agent at a weight ratio of 10:0.5 (prepolymer:curing agent). A total of 1 mL of this mixture was poured onto a microscope glass slide (75 × 26 × 1 mm). Air bubbles were removed under vacuum for 20 min. This mixture was baked at 75 °C for 2 h to cure the PDMS and form a thin film. A thin layer (∼50 *μ*m) of additional uncured PDMS mixture was spread on this cured PDMS film (∼1 mm) to form a sticky layer. Sand was sifted through a 200 *μ*m mesh strainer onto this sticky layer of the film to partially embed the sand in the sticky uncured PDMS layer. The sand-PDMS film was then baked at 75 °C for 30 min to cure the sticky layer and ensure the sand was adhered to the PDMS film. Afterwards, the sand-PDMS film was removed from the glass slide using a blade, cut into 3 mm × 5 mm pieces as the final devices, and attached onto the inside wall of a cryovial with the sand facing the inside of the cryovial. Finally, the sand-PDMS film containing cryovials were autoclaved at 121 °C for 30 min before use for cell cryopreservation.

### Measurement of Ice-Seeding Temperature.

To measure the ice-seeding temperature initiated by the sand-PDMS film, a piece of sand-PDMS film was attached to the inside wall of a 2-mL glass vial, and 1 mL of cryopreservation solution was added into the vial (*n* = 6 independent runs). The cryopreservation solution contained 1.5 M dimethysulfoxide (DMSO), 0.1 M sucrose, and 10% fetal bovine serum (FBS) (Sigma-Aldrich) in the L15 medium. Glass vials without a sand-PDMS film and with the same volume of cryopreservation solution were used as a control. A K-type thermocouple (Omega, Norwalk, CT, 0.05 in. in diameter) was then placed in the cryopreservation solution in the glass vial. The vials were placed onto the shelf of a SP Scientific (Warminster, PA) Virtis AdVantage Pro benchtop lyophilizer and precooled to 4 °C. The shelf temperature was then programed to cool to −35 °C with a 40 min ramp time, resulting in a cooling rate of ∼1 °C/min, to slowly cool the samples. The thermocouple was connected to a Keysight Technologies (Santa Rosa, CA) 34970A Data Acquisition System to record the temperature over time. The formation of ice in the sample was noted by a sudden increase in temperature during cooling, due to the latent heat release associated with ice formation. The temperature when this sudden increase occurred was recorded as the ice-seeding temperature.

### Isolation of Ovarian Follicles.

Ovaries were obtained from 3 to 8-week-old C57BL/6 mice (The Jackson Laboratory, Bar Harbor, ME). All animal procedures and studies were approved by the Institutional Animal Care and Use Committee (IACUC #R-APR-21-22) at the University of Maryland, College Park, MD. Preantral secondary follicles (100–150 *μ*m in diameter) were isolated from ovaries using a cell dissociation sieve (Sigma-Aldrich) that was first proposed for follicle isolation from domestic cat ovaries [32] and has more recently been shown to yield similar results to mechanical isolation via syringes for mouse follicle isolation [33]. Briefly, ovaries were placed on the metal mesh of the cell dissociation sieve and gently ground using the accompanying glass pestle over top follicle dissection medium (L15 supplemented with 1% penicillin/streptomycin and 1% FBS) on a heated stage (37 °C). Size 60 mesh (Sigma-Aldrich) was used for the procedure (wire diameter: 0.254 mm; opening size: 380 *μ*m). This procedure took less than 10 min to homogenize the tissue and release preantral ovarian follicles. Follicles were manually selected under a stereomicroscope and transferred to fresh, prewarmed follicle dissection medium for washing. The criteria for selecting follicles were ∼100–200 *μ*m in diameter that appeared morphologically normal under stereomicroscope, meaning: round (no flattening) in overall follicle shape, round oocyte in the center without obvious damage (i.e., no darkening or flattening), intact follicular basement membrane, at least one layer of granulosa cells, without visible damage or large separation of granulosa cells from oocyte. Follicles were then transferred to recovery media (αMEM supplemented with 1% penicillin/streptomycin and 10% FBS) and allowed to recover in a 37 °C incubator for 2 h before further experiments.

### Cryopreservation of Ovarian Follicles.

Follicles were cryopreserved using a slow-freezing procedure with a highly affordable Mr. Frosty™ freezing container filled with isopropyl alcohol (Sigma-Aldrich). Isolated follicles were transferred into precooled (4 °C) cryopreservation solution and allowed to equilibrate for 30 min on ice. Then, follicles (approximately 10–20 per group for each independent experiment) were transferred into 1 mL of fresh precooled cryopreservation solution in a cryovial either with or without a sand-PDMS film. The cryovials were placed into the Mr. Frosty™ and stored in a −80 °C freezer overnight. This generated a cooling rate of ~1 °C/min to freeze the sample. Then, the cryovials were transferred into liquid nitrogen (LN2) for long-term storage (1–4 weeks).

### Thawing of Ovarian Follicles.

The cryovials containing follicles were removed from LN2 storage and warmed by plunging into a 37 °C water bath with gentle shaking until the sample was completely thawed. The cryopreservation solution was diluted in a stepwise matter to minimize cell damage and preserve cell function [11]: Follicles were transferred into a 35 mm Petri dish with 3 mL of L15 medium supplemented with: (1) 1 M DMSO + 0.1 M sucrose + 10% FBS for 10 min; (2) 0.5 M DMSO + 0.1 sucrose + 10% FBS for 10 min, (3) 0.1 M sucrose + 10% FBS for 10 min; and (4) 10% FBS for 5 min. Follicles were then transferred into recovery media (αMEM + 1% penicillin/streptomycin + 1% FBS) and incubated at 37 °C with 5% CO_2_ for 2 h before further use.

### Immediate Viability of Follicles.

After incubation in the recovery media for 2 h, follicles were stained with calcein AM and propidium iodide (PI) to visualize live (green stain) and dead (red stain) cells, respectively, according to the instructions of the manufacturer (ThermoFisher). The two dyes were added into recovery media (1 *μ*M for calcein AM and 1 *μ*g mL^−1^ for PI) and incubated with the follicles for 15 min at 37 °C. Afterwards, green and red fluorescence images were taken using a Zeiss (Oberkochen, Germany) LSM710 microscope to quantify the green (live) and red (dead) areas of the follicles. The green (live) and red (dead) areas were measured as a percentage of the total area of the follicle taken from the morphology image. The viable area of the follicle was calculated by subtracting the red area from the total area, unless the green area was less than this value, then the green area was used as the viable area value. In other words, the viable follicle area was the follicle area that had esterase activity measured by the calcein AM and no damage measured by the PI. Immediate cell viability was calculated as the percentage of follicles with a viable follicle area (green stain only, not overlapping with red stain) greater than 70% of the total follicle area. Freshly isolated follicles were examined in the same way as positive control.

### In Vitro Culture of Follicles.

For in vitro culture of follicles, they were encapsulated in 0.25% alginate (w/v) beads using a pipette droplet method as previously reported [34,35]. The 0.25% alginate solution was composed of PRONOVA UP MVG sodium alginate (NovaMatrix, Sandvika, Norway) dissolved in a 25 mM D-mannitol and 10 mM HEPES solution and sterile-filtered through a 0.22 *μ*m filter. Follicles were first transferred via pipette into a ∼50 *μ*L alginate solution droplet for washing. Then, follicles were transferred via pipette to a fresh ∼50 *μ*L alginate solution droplet. A small volume pipette (10 *μ*L) was used to form 5 *μ*L alginate droplets containing one follicle each. The pipette was used to take up ∼2.5 *μ*L alginate, then the follicle, then the rest of the ∼2.5 *μ*L alginate, so that the follicle was close to the center of the bead. This 5 *μ*L alginate droplet containing one follicle was ejected into a crosslinking solution consisting of 50 mM CaCl_2_ and 140 mM NaCl [34]. The alginate beads were crosslinked in the crosslinking solution for 2 min and then transferred to recovery medium for washing. Then, the follicle-laden alginate beads were transferred to a 96-well plate, with each well containing one follicle-laden alginate bead and 150 *μ*L of follicle culture media was added to each well. Follicle culture medium consisted of αMEM supplemented with 1x insulin-transferrin-selenium solution (Sigma-Aldrich), 1% L-glutamine, 1% penicillin/streptomycin, 10% FBS, and 100 mIU/mL follicle stimulating hormone (Sigma-Aldrich). Follicles were cultured at 37 °C with 5% CO_2_ for 9 days. The day of alginate encapsulation was considered day 1. Every other day, 75 *μ*L of the follicle culture medium was manually replaced with fresh media.

Using a Zeiss LSM710 microscope, follicles were imaged on days 1, 3, 5, 7, and 9. Follicle diameters were measured using ImageJ (1.52q, U.S. National Institutes of Health, Bethesda, MD). To account for varying follicle shapes (e.g., spheroidal), two measurements were averaged to give the reported follicle diameter, with one measurement at the widest length of the follicle and the other perpendicular to it across the follicle. Growth was assessed by the change in the follicle diameter, *D*/*D*1, which is the follicle diameter over the initial follicle diameter on day 1. Follicles with a *D*/*D*1 greater than 1 on day 9 were characterized as growing, surviving follicles. A *D*/*D*1 less than 1 signaled that a follicle that was dying, degenerating, and shrinking. Freshly isolated follicles were studied in the same way as positive control.

### Statistical Analysis.

All graphing and statistical analyses were performed using prism 8 (GraphPad Software, San Diego, CA). Data are reported as the mean±standard deviation from at least three independent runs. Two-group comparisons were performed using Student's two-tailed unpaired *t*-test assuming equal variance. For comparisons between multiple groups with one independent variable/factor, a one-way analysis of variance (ANOVA) followed by a Tukey post hoc correction was performed. For comparisons between groups with two variables/factors, two-way ANOVA with Sidak's post-test and correction was used. The differences were considered statistically significant when the *p* value was less than 0.05 (*p <* *0.05, **p <* *0.01, and ***p <* *0.001).

## Results

**Fig. 1:**
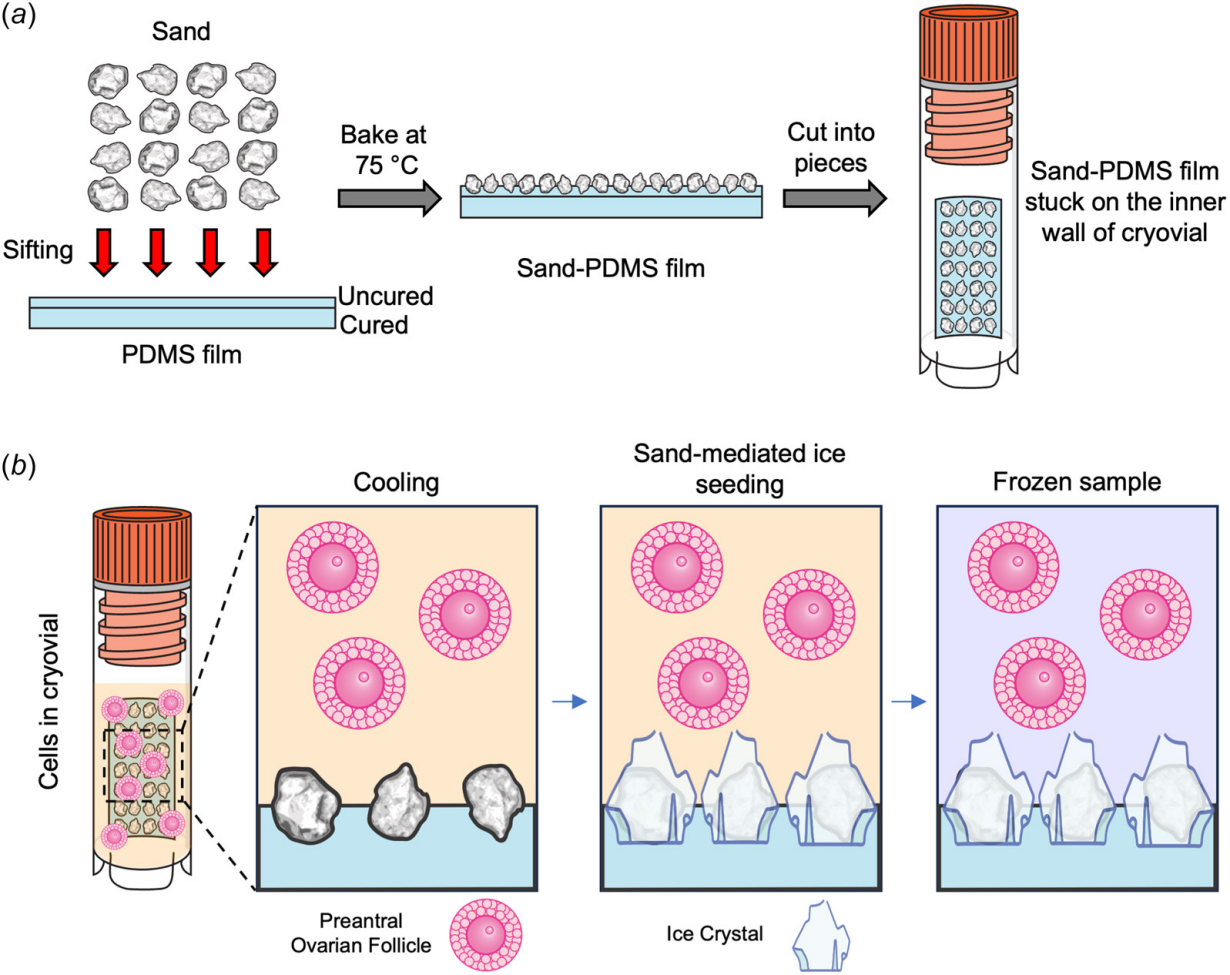
Preparing and using the sand-PDMS film device for cryopreservation. (*a*) A schematic illustration for preparing the sand-PDMS film and attaching it to the inside wall of a cryovial. Sand is first washed and sterilized before being sifted through a 200 *μ*m pore size mesh strainer onto the PDMS film. The PDMS film has a thin layer of uncured PDMS on top, acting like a sticky glue to immobilize the sand particles on its surface. After baking at 75 °C to cure the PDMS layer and fully secure the sand particles onto the film, the sand-PDMS film is cut into smaller pieces (3 mm × 5 mm) and attached to the inner wall of a cryovial, with the smooth size of the PDMS attached to the wall and the sand-laden side exposed to the inside/sample. (*b*) A schematic illustrating the ice nucleation of the sand-PDMS film during slow-freezing cryopreservation. Preantral ovarian follicles in cryopreservation solution are added into the cryovial with the sand-PDMS film. During cooling, sand initiates the formation of ice crystals in the extracellular space, near the sand particles immobilized on the PDMS film and ice then propagates into the whole sample.

This study investigates the use of sand (partially embedded in a PDMS film), a natural, biocompatible ice nucleator, with a slow-freezing protocol for cryopreservation of preantral ovarian follicles. We created the sand-PDMS film devices to attach on the wall of a cryovial to nucleate ice during slow-freezing cryopreservation of ovarian follicles. First, the sand is partially embedded in a PDMS film that has a thin, uncured top layer of PDMS to act as a glue for the sand particles, as seen in Fig. 1(*a*). After baking in a 75 °C oven to cure the uncured, “glue” layer of PDMS, the sand-PDMS film is created with the sand particles being immobilized in the PDMS. This film is then cut into smaller pieces (3 mm × 5 mm) that can be attached to the inside wall of the cryovial via the smooth PDMS surface without sands. This allows for the exposed sand particles to come into contact with the sample and nucleate ice in the extracellular solution, but still remain in the PDMS film on the wall of the cryovial without contaminating the sample. Preantral ovarian follicles in cryopreservation solution are added into the cryovial, where the sand on the PDMS film causes ice crystals to nucleate and the sample to freeze (Fig. 1(*b*)).

### Sand-Polydimethylsiloxane Film Increases the Temperature of Ice-Seeding in the Cryopreservation Solution.

**Fig. 2:**
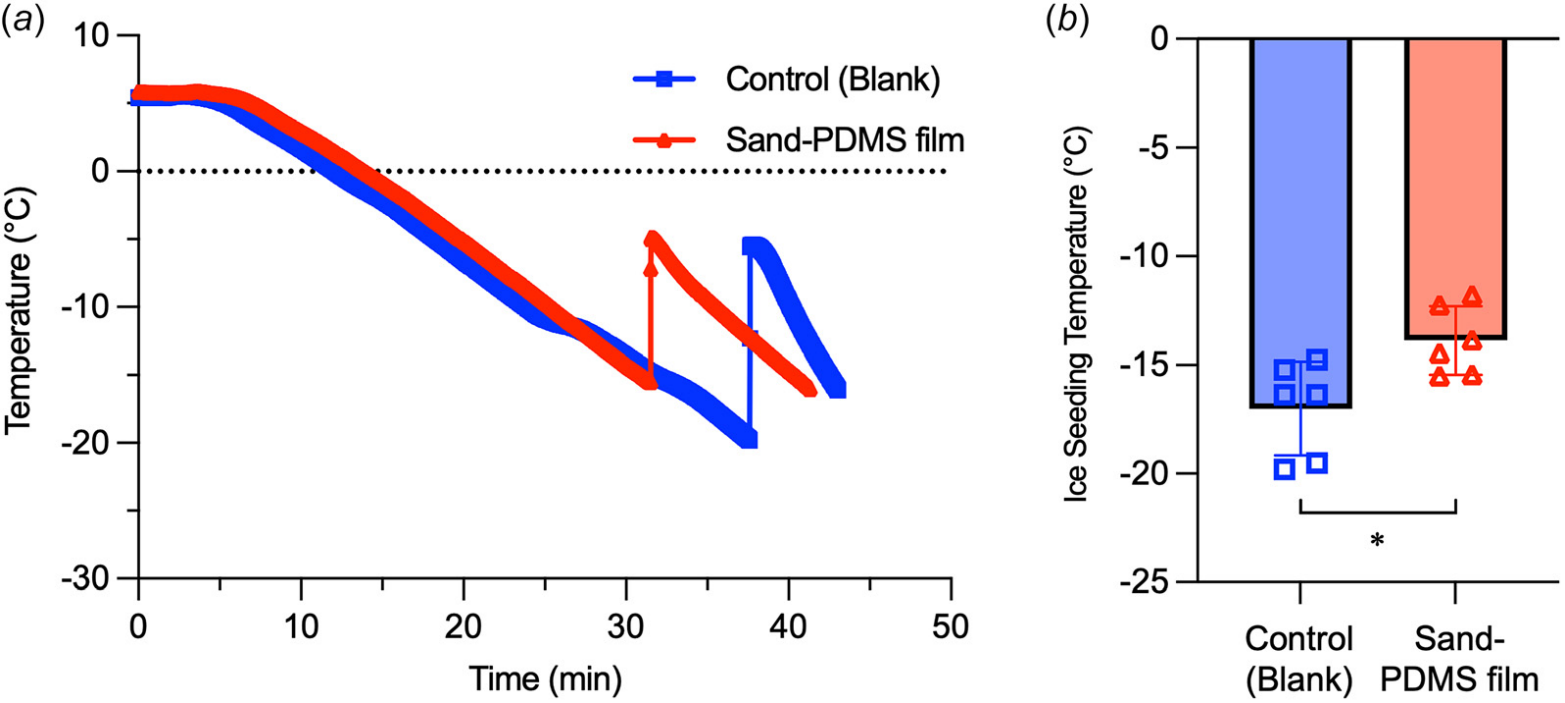
High subzero ice-seeding temperature is enabled by the sand-PDMS film. (*a*) Representative thermal histories of follicle cryopreservation solution during slow-cooling of samples with no film (control (blank)) and with sand-PDMS film (sand-PDMS film). The ice-seeding temperature is indicated by a sudden increase in temperature due to the release of latent heat of liquid water in the sample changing phase into ice. (*b*) Quantitative data of the ice-seeding temperature in follicle cryopreservation solution under the aforementioned two conditions (*n* = 6 independent runs). *p <* *0.05. Student's two-tailed unpaired *t*-test assuming equal variance was used for statistical analysis.

The effect of sand on the ice-seeding temperature of the cryopreservation solution (10% DMSO + 0.1 M sucrose + 10% FBS in L15 medium) for follicle cryopreservation was investigated by measuring the change in temperature over time during cooling. The initiation of ice formation in the solution can be detected by a sudden temperature rise, due to the release of latent heat of fusion caused by ice nucleation and growth. The temperature at which this sudden increase occurs is defined here as the ice-seeding temperature. Figure 2(*a*) shows a representative thermal history of the cryopreservation solution without any film (control) and with a sand-PDMS film during cooling, with Fig. 2(*b*) showing the quantitative results for the ice-seeding temperature of the solution in both conditions. During slow cooling (∼1 °C/min) similar to that experienced in a Mr. Frosty, ice nucleates at an average temperature of −17.0±2.2 °C with some trials showing ice formation as low as −20 °C in the absence of a sand-PDMS film, which can be deadly to cells. When the sand-PDMS film is added to the cryopreservation solution, the ice-seeding temperature shows a statistically significant increase to −13.9±1.6 °C. It has been previously shown that the PDMS film itself (without any sand particles immobilized on the surface) does not significantly impact the ice-seeding temperature [31].

### Sand-Mediated Ice-Seeding Enhances Immediate Viability of Cryopreserved Ovarian Follicles.

**Fig. 3:**
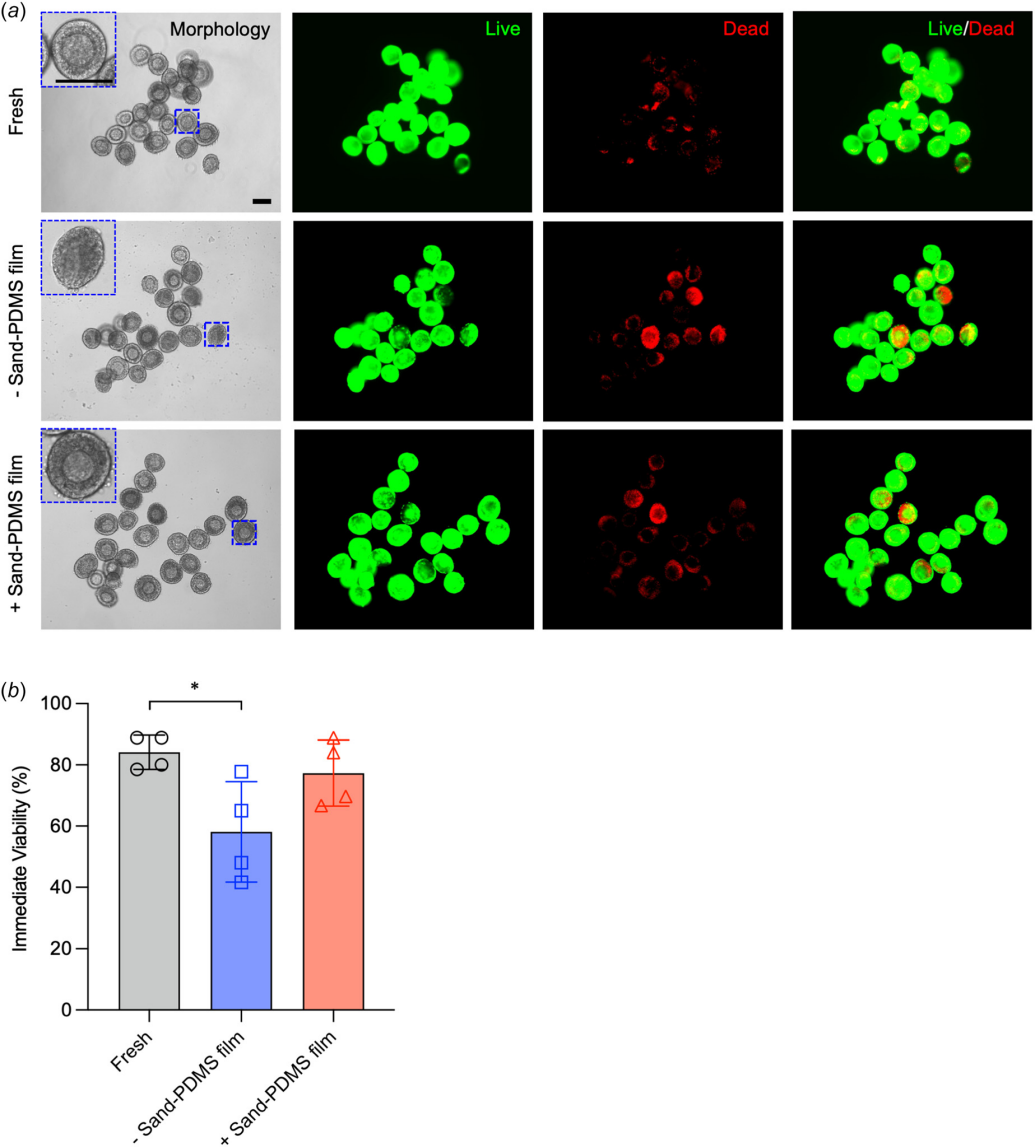
Immediate viability of fresh and cryopreserved ovarian follicles. (*a*) Immediate (after 2 h incubation in recovery medium at 37 °C) viability of fresh ovarian follicles, follicles cryopreserved without using the sand-PDMS film, and follicles cryopreserved using the sand-PDMS film assessed via live/dead (green/red in second/thrid column) staining. The cryopreservation solution contains 10% DMSO, 10% FBS, and 0.1 sucrose in L15 medium. The dashed box shows a zoom-in image of a single follicle in the sample. Scale bar = 100 *μ*m. (*b*) Quantitative data of the immediate viability of ovarian follicles. Follicle was assessed by the viable follicle area (area staining only green, not green and red). Follicles with a viable area of more than 70% are considered viable, and immediate viability is calculated as the number of follicles with a viable area of more than 70% over the total number of follicles in each independent run (*n* = 4). *p <* *0.05. One-way ANOVA followed by a Tukey post hoc correction was used for statistical analysis.

The effect of the increased ice-seeding temperature mediated by the sand-PDMS film was explored for cryopreservation of preantral ovarian follicles by slow-freezing. Secondary ovarian follicles (size 100–150 *μ*m) were cryopreserved in follicle cryopreservation solution with or without the addition of a sand-PDMS film on the wall of the cryovial and stored in LN2 for 1–4 weeks. After thawing, the immediate viability was assessed via live/dead staining, with representative images shown in Fig. 3(*a*), where live cells are stained green (second column) and dead cells are stained red (third column). Fresh follicles (isolated and not cryopreserved) were also stained as a positive control. Morphologically normal and healthy secondary follicles isolated from ovarian tissue are identified as those containing a central, round oocyte surrounded by multiple layers of follicular cells. Freshly isolated follicles display normal morphology and high viability per live/dead staining (84.1±5.6% of follicles with >70% of the total follicle area being in green staining; Figs. 3(*a*) and 3(*b*)). There are some dead cells in the isolated follicles, most likely due to the isolation process causing some membrane damage to parts of the follicles. Many follicles cryopreserved without the sand-PDMS film also display healthy morphology and live/green staining, but cryodamage to the follicles is evident via the increase of red staining in the follicles, especially seen in the four follicles with bright and extensive red staining in Fig. 3(*a*), compared to the fresh control. Cryopreservation without the sand-PDMS film results in a significant decrease in viability (58.1±26.4%) compared to both the freshly isolated control and cryopreservation using the sand-PDMS film (77.3±10.8%), as displayed in Fig. 3(*b*). There is no significant difference between the immediate viability of the freshly isolated control and the sand-PDMS film groups. There are also more follicles in the cryopreservation group without the sand-PDMS film than the fresh group that show damaged follicle morphology, as represented in the blue dashed inset, whereas follicles cryopreserved using the sand-PDMS film more closely resemble the fresh follicles. These data of the immediate viability show a modest effect of the controlled ice-seeding on immediate cell viability via the sand-PDMS film.

### Sand-Mediated Ice-Seeding Enhances In vitro Growth of Cryopreserved Ovarian Follicles.

**Fig. 4:**
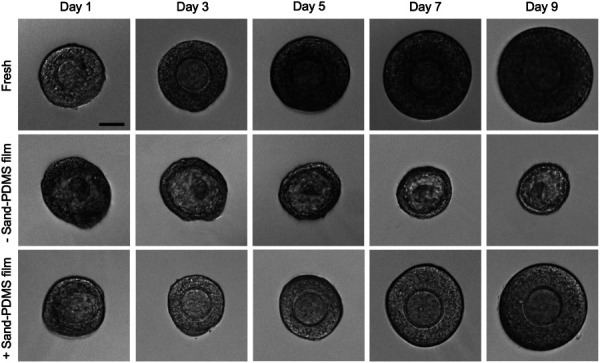
Representative morphology of fresh and cryopreserved ovarian follicles during in vitro culture. Follicles cryopreserved with or without the sand-PDMS film were thawed and encapsulated in 0.25% alginate for 9 days of in vitro culture. Fresh follicles were encapsulated on the same day of isolation as a control. Images of the follicle were taken every other day of culture, starting on day 1. Scale bar = 50 *μ*m.

To further evaluate follicle quality beyond immediate viability, which, based on the live/dead staining assay, mainly reflects the cell membrane integrity measured by PI and esterase activity measured by the calcein-AM dye, cryopreserved follicles were cultured in vitro to assess follicle growth (an indicator of long-term viability) over time. Follicles cryopreserved with or without the sand-PDMS film were encapsulated in 0.25% alginate beads after thawing for three-dimensional culture, and the change in diameter was measured over 9 days of culture to quantify growth. Freshly isolated follicles were also cultured in the same way as a control. All follicles chosen for cryopreservation and post-thaw in vitro culture showed similar morphology prior to cryopreservation. Figure 4 shows representative images of the follicles from the three culture groups—(1) freshly isolated follicles (Fresh), (2) follicles cryopreserved without the sand-PDMS film (− sand-PDMS film), and (3) follicles cryopreserved with the sand-PDMS film (+ sand-PDMS film)—on every other day of culture when diameter measurements were made. Most of the fresh follicles maintain the round morphology and intact oocyte through day 9 in culture and grow in size, indicating healthy, properly functioning follicles. Most of the follicles cryopreserved without the sand-PDMS film show signs of damage on day 1 of culture, with irregular morphology and a darkened oocyte. During culture, the follicles undergo atresia and shrink in size, and their darkened oocytes become barely visible in 9 days. Most of the follicles cryopreserved with the sand-PDMS film show round morphology similar to the fresh control on day 1, with the oocyte clearly visible in the center of the follicle and normal surrounding layers of granulosa cells. The follicles maintain the round morphology during culture and grow in size similar to the fresh control in 9 days.

**Fig. 5:**
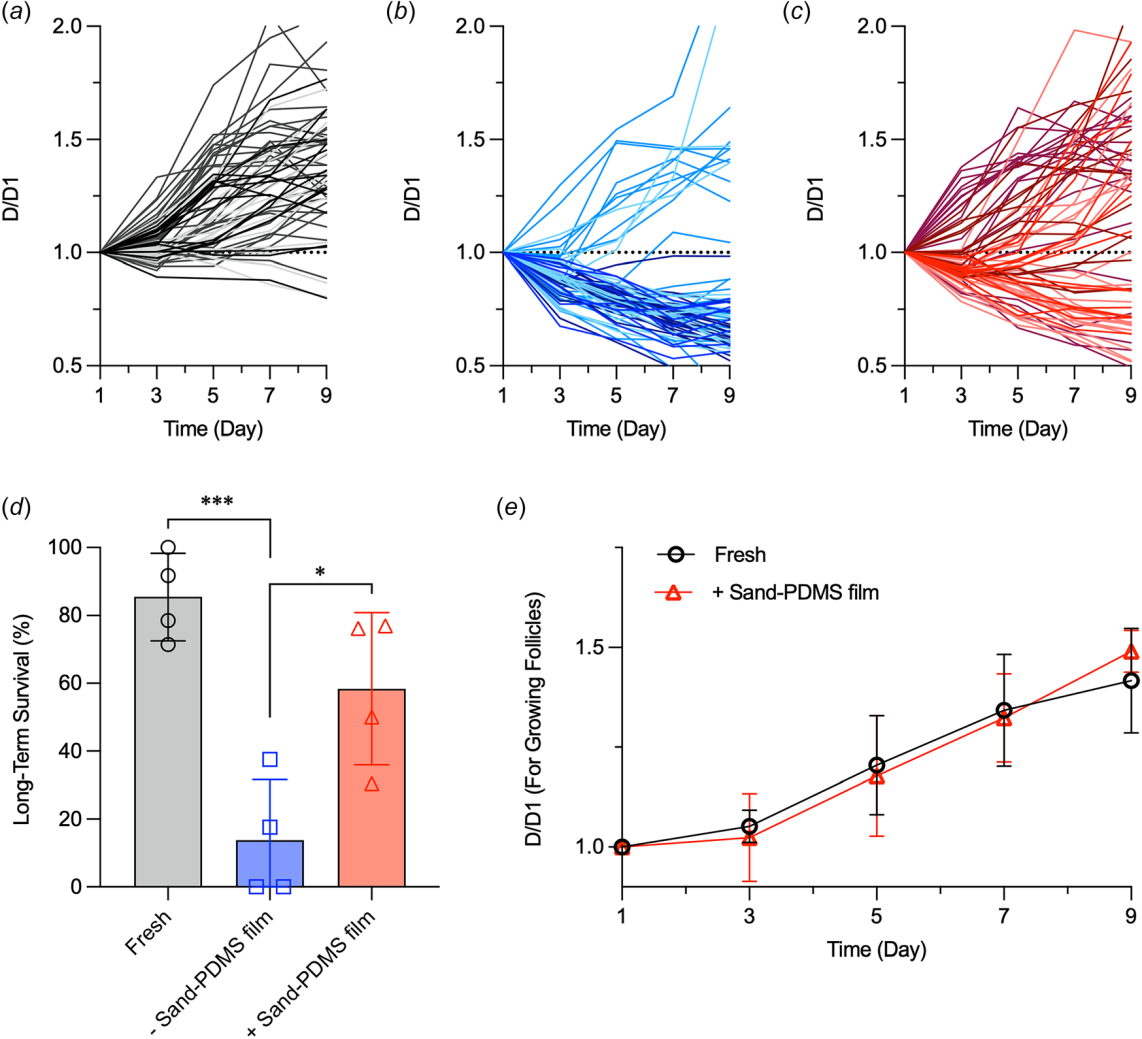
Growth and long-term survival of fresh and cryopreserved ovarian follicles during in vitro culture. Individual growth curves of (*a*) freshly isolated follicles, (*b*) follicles cryopreserved without the sand-film, and (*c*)follicles cryopreserved using the sand-film during 9 days of in vitro culture (*n* = 4 independent runs). Each curve represents the growth of one individual follicle. Each independent run is represented by a different shade of the experimental group's color. The growth of each follicle is evaluated by the diameter of the follicle (*D*) over time normalized to the diameter of the follicle on day 1 (*D*1). A *D*/*D*1 ratio over 1 means the follicle is growing in diameter, while a *D*/*D*1 ratio less than 1 signifies that a follicle is shrinking and dying during the 9 days of culture. (*d*) Survival of the ovarian follicles after 9 days of culture. The survival is calculated as the percentage of the number of follicles with a *D*/*D*1 greater than 1 on day 9 out of the total number of follicles in culture (*n* = 4 independent runs). One-way ANOVA followed by a Tukey post hoc correction was used for statistical analysis. (*e*) Mean growth curves of all surviving follicles as a whole from the fresh and sand-film cryopreservation groups during 9 days of in vitro culture (*n* = 4 independent runs). *p <* *0.05 and ***p <* *0.001. Two-way ANOVA with Sidak's post-test and correction was used for statistical analysis.

To quantify and compare the growth of individual follicles during culture, the diameter of the follicle on every other day of culture was measured and normalized to the original diameter on day 1 (*D*1) of culture. Follicle growth was thereby quantified by the ratio *D*/*D*1, where *D* is the diameter of the follicle over time. A *D*/*D*1 ratio greater than 1 indicates that the follicle is growing, while a *D*/*D*1 ratio less than 1 indicates that a follicle is not growing and dying during the 9 days of culture. In the fresh follicle group, the majority (52/60) of the follicles grow (up to two times the original size) by day 9 (Fig. 5(*a*)). For the follicles cryopreserved without the sand-PDMS film, most (58/70) of the follicles show a *D*/*D*1 less than 1 (Fig. 5(*b*)), indicating most of the follicles are damaged by the cryopreservation process and do not survive in long term. For the follicles cryopreserved using the sand-PDMS film, the majority (40/71) show *D*/*D*1 values over 1 during culture (Fig. 5(*c*)), with some follicles showing a *D*/*D*1 ratio of up to 2 on day 9 similar to the fresh controls. However, there are a noticeable number of follicles that are damaged by the cryopreservation process and fail to grow in culture, as evidenced by the *D*/*D*1 values of less than 1 during the culture period.

This diameter ratio was used to quantify the number of surviving follicles in each condition during in vitro culture, and follicles with a *D*/*D*1 ratio greater than 1 on day 9 are considered viable. The long-term survival is quantified as the number of viable follicles out of the total number of follicles cultured for each experimental group. As shown in Fig. 5(*d*), follicles cryopreserved with the sand-PDMS film have a similar long-term survival rate compared to the fresh control, with no statistically significant difference (58.4±22.5% versus 85.4±12.8%). However, without the sand-PDMS film present during the cryopreservation process, the long-term follicle survival drops to 13.8±17.9%. Although the difference in the long-term viability between the fresh follicles and follicles cryopreserved with the sand-PDMS film is not statistically significant, the follicles cryopreserved using the sand-PDMS film have a lower average long-term survival and a higher standard deviation. Nonetheless, there is still some damage to the follicle sample during the cryopreservation process with the sand-PDMS film, which could motivate further improvement of the cryopreservation solution composition and/or CPA concentration to protect the cells during the freezing/thawing process.

Comparing the growth of all the follicles surviving the entire 9-day culture period as a whole in each run from four independent runs of the fresh and sand-PDMS film groups (Fig. 5(*e*)), follicles in both groups are able to grow up to 1.5 times of their original diameter. There is no significant difference between the growth (*D*/*D*1 ratio) of surviving follicles from the freshly isolated group and + sand-PDMS film groups on any day of in vitro culture, indicating that cryopreservation using the sand-PDMS film to nucleate ice does not cause lasting damage to the surviving ovarian follicles.

## Discussion

The cryopreservation of ovarian tissue is an essential technology for preserving a patient's fertility. While it is more common to cryopreserve sections of cortical tissue from the ovary and later transplant the cortical fragments [36], cryopreserving isolated preantral ovarian follicles offers distinct advantages over tissue. Because preantral ovarian follicles are small (typically less than 150 *μ*m in diameter), the issue of CPA perfusion that causes impairment to larger tissues is mitigated [37]. Transplantation of cryopreserved ovarian tissue has also been shown to result in extensive follicle loss due to ischemia-reperfusion issues [38], which could be resolved by either grafting individual follicles in an artificial ovary environment [39,40] or culturing and maturing follicles in vitro for in vitro fertilization [41]. In cases where malignant cells may be present in the vascularized ovarian tissue due to some types of cancer, cryopreservation of isolated follicles avoids the risk of re-introducing these malignant cells back into the patient during transplantation [42], as the follicles have their own membrane which prevents them from coming into contact and being infected with the malignant cells [43].

Current cryopreservation of ovarian tissue/follicles is done by one of two methods: vitrification or slow-freezing [44]. While vitrification is widely studied for oocytes [45] and has shown success for preantral ovarian follicle cryopreservation [46,47], it typically requires a high concentration of toxic CPA to achieve ice-free cooling of the sample. Slow-freezing is convenient and has been used to successfully cryopreserve preantral ovarian follicles with high survival/viability (∼70%) [11,48]. However, the slow-freezing protocol used in these studies required a programmable-rate freezer to hold the sample at a high subzero temperature so that ice could be manually seeded in the sample by touching the cryovial with forceps (or presumably another metal tool) prechilled in LN2. Controlling the temperature of ice nucleation is critical for ovarian tissue samples [25,49,50]. Out of the four trials for the follicles cryopreserved without the sand-PDMS film, two trials did not have any surviving follicles after 9 days of culture, while the other two trials had a survival of 20–40% on day 9 (Fig. 5(*b*)). This may be due to the uncontrolled stochastic ice nucleation during the cryopreservation process; some trials may have had much lower temperatures of ice nucleation, resulting in increased damage to the follicles, while the other trials may have had ice nucleated at higher subzero temperatures.

The sand-PDMS film is able to control ice nucleation in the cryopreservation solution (Fig. 2), increasing the ice-seeding temperature to −13.9±1.6 °C compared to the blank control (−17.0±2.2 °C). The ice-seeding temperatures reported here are lower than those reported for sand in pure water: the ice-seeding temperatures of water with or without the sand-PDMS film were reported as −7.8±1.6 and −15.9±1.6, respectively [31]. This is most likely due to the presence of CPA in the cryopreservation solution tested: penetrating CPAs, like DMSO, can depress the freezing point of solutions due to colligative properties (adding CPAs reduces the chemical potential of extracellular water) [51] and interference with the hydrogen bonding network of water molecules [52,53]. Additionally, the amount of sand embedded onto the PDMS film was sufficient to achieve consistent ice nucleation at high subzero temperatures, as evidenced by the small standard deviation and consistent ice-seeding temperature measured in Fig. 2.

Because the sand-PDMS film is able to consistently seed ice at high subzero temperatures, there are no trials where the follicles cryopreserved with the sand-PDMS film are all damaged/dead. Follicles cryopreserved here using sand-mediated ice-seeding at high subzero temperatures show improved immediate viability (77.3±10.8%) and long-term survival (58.4±22.5%) that are not significantly different from that of fresh follicles (84.1±5.6% and 85.4±12.8% for immediate and long-term viability, respectively, Figs. 3(*b*) and 5(*d*)), without the added cost of the programmable-rate freezer and the manual hassle of ice-seeding using similar concentration of CPA. However, there is still room to further improve the cryopreservation protocol in view of the larger variation in the long-term survival of the cryopreserved than fresh follicles. For example, this can be done via optimizing the solution for cryopreservation in a future study. The sand-PDMS film is simple and easy to add to the cryovial wall, allowing the use of a highly affordable device (Mr. Frosty™) for cooling in this cryopreservation procedure. It is worth noting that the standard practice of using the Mr. Frosty™ device for cryopreservation is that no manual ice-seeding is done, because the device does not have the function of measuring temperature or holding the sample at a specific temperature for ice-seeding. This is why a control group of manual ice-seeding was not included in this study.

The difference in viability/survival of the tested conditions between the immediate viability experiment (2 h post-thaw) and the in vitro culture experiment (up to 9 days post-thaw) shows that immediate viability via live/dead staining is not sufficient for assessing the quality of cryopreserved follicles. Although the trends for both assessments are the same, with the fresh follicles and follicles cryopreserved with the sand-PDMS film outperforming the follicles cryopreserved without the sand-PDMS film, the values for the follicles cryopreserved without the sand-PDMS film are drastically different. Based on the live/dead staining quantification, more than half (58.1±16.4%) of the follicles cryopreserved without sand are viable, whereas only 13.8±17.9% are able to survive and grow in culture. On the day of thawing, there is not a drastic difference in the morphology of follicles cryopreserved with or without the sand-PDMS film, as most appear round and intact with similar levels of live and dead staining. However, the morphologies of the two groups greatly differ on day 1 of culture, as most of the follicles cryopreserved without the sand-PDMS film show clear evidence of cryodamage.

Finally, the ease of this method allows for possible combination with other technologies investigated to improve cryopreservation outcomes. Encapsulation of preantral ovarian follicles in alginate hydrogels helps to preserve the three-dimensional structure of the follicle and allows for growth and development in vitro [54–57]. Alginate hydrogel encapsulation has also been shown to enhance cryopreservation outcomes [18,46,58], so encapsulating the follicles before slow-freezing could be combined with the sand-mediated ice-seeding method. Using an alternative, nontoxic CPA, like trehalose, delivered into the preantral ovarian follicles using cold-responsive nanoparticles [59] could help reduce or even eliminate the use of DMSO and other possibly toxic, penetrating CPAs.

## Conclusions

This study examined the use of a natural, biocompatible material—sand—as an ice nucleator during slow-freezing cryopreservation of preantral ovarian follicles. The quality of the ovarian follicles after thawing was assessed via live/dead staining and in vitro culture, showing that the follicles cryopreserved using the sand retained high viability and capacity to grow after thawing. This study validates the effectiveness of using the sand mediated ice-seeding method for cryopreservation of multicellular microtissue and further demonstrates the ease with which it can be incorporated into existing slow-freezing protocols. Using sand as an ice nucleator can eliminate the need for costly programmable-rate freezers and highly user-dependent manual ice-seeding, making slow-freezing an attractive and reliable method for preserving ovarian follicles as an alternative for preserving fertility.

## Funding Data

- Maryland Stem Cell Research Fund (MSCRF, 2023-MSCRFV-6014; Funder ID: 10.13039/100012443).
- National Science Foundation Graduate Research Fellowship Program (NSF GRFP, DGE 1840340; Funder ID: 10.13039/100000001).
- National Institutes of Health (NIH R01EB023632; Funder ID: 10.13039/100000002).

## Conflict of Interest

X.H. filed patent application for the controlled ice-seeding technology reported in this work through the University of Maryland, College Park, and is licensing the technology to his startup company HOHCells, LLC.

## Data Availability Statement

The datasets generated and supporting the findings of this article are obtainable from the corresponding author upon reasonable request.
